# Weather Conditions and Maturity Group Impacts on the Infestation of First Generation European Corn Borers in Maize Hybrids in Croatia

**DOI:** 10.3390/plants9101387

**Published:** 2020-10-18

**Authors:** Renata Bažok, Ivan Pejić, Maja Čačija, Helena Virić Gašparić, Darija Lemić, Zrinka Drmić, Martina Kadoić Balaško

**Affiliations:** 1 Department of Agricultural Zoology, University of Zagreb Faculty of Agriculture, Svetošimunska Cesta 25, 10000 Zagreb, Croatia; rbazok@agr.hr (R.B.); hviric@agr.hr (H.V.G.); dlemic@agr.hr (D.L.); mbalasko@agr.hr (M.K.B.); 2 Department of Plant Breeding, Genetics and Biometrics, University of Zagreb Faculty of Agriculture, Svetošimunska Cesta 25, 10000 Zagreb, Croatia; ipejic@agr.hr; 3 Croatian Agency for Agriculture and Food, Plant Protection Center, Vinkovačka Cesta 63c, 31000 Osijek, Croatia; zrinka.drmic@hapih.hr

**Keywords:** *Ostrinia nubilalis*, tolerance, correlation, *Zea mays*, FAO maturity groups, damage

## Abstract

Overwintering success and weather conditions are the key factors determining the abundance and intensity of the attack of the first generation of European corn borers (ECB). The tolerance of maize to the 1st generation of ECB infestation is often considered to be connected with the maize maturity time. The aims of this research were (I) to examine the reactions of different maize FAO maturity groups in term of the damage caused by ECB larvae, (II) to analyze the influence of four climatic regions of Croatia regarding the damage caused by ECB larvae, and (III) to correlate observed damage between FAO maturity groups and weather conditions. First ECB generation damage has been studied in the two-year field trial with 32 different hybrids divided into four FAO maturity groups (eight per group) located at four locations with different climatic conditions. The results showed a lack of correlation between the FAO maturity group and the percent of damage. The percent of damage was positively correlated with the average air temperature in June (r = 0.59 for 2017 and r = 0.74 in 2018, *p* = 0.0001) within the range from 20 to 24.5 °C and was negatively correlated with the relative air humidity (r = −0.58 in 2017 and r = −0.77 in 2018, *p* = 0.0001) within the range of 50% to 80%. Our results provide a better understanding of the different factors that influence ECB damage. The obtained data could be used to predict the damage from the first generation of ECB under the weather conditions of different regions.

## 1. Introduction

The European corn borer (ECB) (*Ostrinia nubilalis* Hübn.), is a serious pest of maize (*Zea mays* L.) in Europe and the United States (US) as well. The pest is native to Europe [1] and was accidentally introduced in the US in 1917 and spread around the country causing serious damage. The losses are estimated at $1 billion per year [2]; hence, ECB is one of the most important pests from an economic point of view. The preferred host plant of this pest is maize [3]; however, ECB is a polyphagous insect and attacks many different plants, including sorghum, pepper, hemp, millet, chrysanthemums, and some weeds [4].

The extent of losses in maize caused by this pest depend on the degree of the infestation, the year, the yield averages and can range from 250–1000 kg/ha [3]. The damage is caused by the larvae that bore into the stems and ears of corn [5]. Leaf feeding and stem tunneling by ECB larvae reduce plant growth and cause stalk lodging and ear dropping, resulting in severe yield losses of up to 30% [6]. Infestation by the first generation of ECB can reduce silage yields by 14% [7]. As a result of the damage, plants become more susceptible to secondary infections caused by different pathogens, such as *Fusarium* spp. or *Ustilago maydis* (DC.) Corda [8,9,10]. This is why Hudon et al. [11] suggested that the pest should be controlled when 40%–50% of the plants have been attacked.

According to Lynch et al., [12] many authors studied yield losses as a result of the damage caused by this pest. They concluded that several different things affect yield losses: the time of infestation, the stage of plant development when the infestation was initiated, and the geographical location. The first research in Croatia conducted by Ivezić [13] showed an average infestation rate of 37% by ECB. During the 1990s, many farmlands in the eastern part of Croatia were abandoned or neglected. Maize stalks were left in the field, which allowed the pest to spread and reproduce without interruption. Consequently, the damages and yield losses have increased. Yield loss in Croatia caused by the ECB range from 2% up to 25% [14]. Research from Ivezić and Raspudić [15] demonstrated that the average infestation rate during the five-year investigation (1992–1996) was 64%. Another research from Raspudić et al. [16] showed that the pest attacked up to 90% of the growing maize in Croatia. These numbers change depending on the climatic conditions, which have an impact on the insect’s growth and development each year.

Many biotic and abiotic factors influence the appearance and intensity of an ECB attack; however, the weather conditions are the most significant ones [17]. The number of generations per year is connected with the climatic conditions. In contrast to the US Corn Belt, where the ECB has up to four generations per year, only one generation is observed in Central Europe [18]. According to Raspudić et al. [19], ECB has two generations in eastern Croatia per year. Daily temperatures and precipitation are very important factors for the ECB population dynamics [20]. We can expect greater damage to maize caused by ECB in a year with increased air temperatures and average precipitation [20].

Moths hatch during May and deposit their eggs on plants at the late whorl stage, before anthesis. The most sensitive stage of this pest to weather conditions is during the egg-laying stage, larva eclosion, and larva first instars. The minimum required temperature for ECB larvae development is 11 °C [21]. According to Rosca and Rada [22], moderate air temperature and high air humidity can result in increased larva eclosion and lower mortality of the first instar larva. On the other hand, high temperatures and drought resulted in higher mortality of ECB first instars larva and low larva eclosion [23]. Heavy storms registered in the period of larvae eclosion can have a negative effect on the population dynamics [24].

Overwintering success together with climatic conditions are the key factors determining the abundance and intensity of the attack of the first generation [25]. Lemić et al. [25], in their research, estimated that 8000 moths/ha can overwinter if a corn field is left unploughed. If one female moth can lay approximately 500 eggs [26] that results in 4 million larvae of the first generation. Thus, destroying severed maize stalks, where the ECB overwinters, is the most important mechanical measure and must be applied to the whole area where maize is grown. Control strategies for reducing yield losses from this insect include planting dates, early harvest, field scouting, using economic thresholds, insecticides, and hybrid resistance or tolerance.

Tolerance is the ability of a maize plant to withstand a certain population density of the insect without economic loss of yield or quality [27]. Yield losses would be much higher if modern maize hybrids did not have some degree of resistance to ECB [28]. Resilience to the ECB of the commercial maize hybrids is now a common feature. Approximately 90% of the 400 maize hybrids on the market have shown a certain degree of resistance in the vegetative phases of development [29]. Alongside resistance, modern maize hybrids are tolerant of a great degree of damage caused by ECB. Hybrid resistance to whorl feeding borers and tolerance to stalk and ear shank tunneling has increased dramatically from 1940s hybrids [30], with some seed companies providing first and second generation corn borer tolerance ratings for their hybrids [31]. The development of tolerant maize hybrids with a strong, robust stalk contributes immensely to reducing yield loss as a consequence of the damage caused by the ECB [32].

Augustinović et al. [33], in their research, recorded differences between Croatian maize hybrids to ECB larvae feeding. ECB larvae prefer to feed on susceptible hybrids and they gain significantly more weight than larvae fed on tolerant hybrids [34]. Additionally, the tolerance to the first generation ECB infestation is very often connected with the maize FAO maturity group. Higher FAO groups of maize have intensive vegetative growth and, hence, a high and robust stems with a large number of big leaves. This is a biological characteristic that attracts the first generation of ECB, and thus, they lay more eggs. A high population level of the first generation could lead to a high level of second ECB generation, which can cause a yield reduction in the hybrids with longer vegetation periods (medium-late FAO maturity groups) [25].

FAO maturity groups may differ in their sensitivity to the first generation of ECB [25,33]. However, the sensitivity is also correlated with weather conditions, in particular with the average daily temperatures, relative air humidity, and the total amount of rainfall in May and June when egg laying and hatching and larval development is expected. Therefore, the aim of this research was to (I) determine the differences among FAO maturity groups regarding the damage caused by ECB larvae, (II) to establish the differences among four climatic regions of Croatia regarding the damage caused by ECB larvae, and (III) to investigate the correlation between the FAO maturity group and climatic factors.

## 2. Results

Statistical analysis of the weather conditions recorded in May and June showed that the temperatures in May and June significantly differed between years (Table 1). The average monthly temperature in May was lower in 2017 compared to 2018, while in June the average monthly temperature was higher in 2017 compared to 2018.

Out of the six weather indicators observed, two of them, the average monthly temperatures in June and the total amount of rainfall in June differed among the locations (Table 2). Šašinovec and Gola were locations with lower temperatures compared to Vrana, and, at the same time, Šašinovec and Tovarnik were locations with higher total amounts of rainfall compared to Vrana.

Although the statistical differences among FAO maturity groups exist for both years of investigation (Table 3), we did not establish any correlation between the FAO maturity group and the percent of the damage caused by the first generation. The correlation coefficients were not significant in both years of investigation (*p* = 0.6561 in 2017 and *p* = 0.3643 in 2018).

The percentage of infestation of each of four FAO groups significantly differs among locations in both years of investigation (Table 4 and Table 5). At the same time, the percentage of infestation significantly differs among FAO groups only once in each year (at only one locality in 2017 and at one locality in 2018).

The highest correlation coefficients (measured by Pearson’s coefficient of correlation) and the highest coefficients of determination (Table 6) were obtained when the percent of infestation was correlated with the mean air temperature and with the average air humidity in June in both years of investigation. According to Roemer-Orphal, established correlations could be described as strong (for the mean air temperature in June in 2017 and 2018 and for the average air humidity in June 2017) or as very strong (for the average air humidity in June 2018). Although the percent of infestation significantly correlated with the total amount of rainfall in June in 2017 and in 2018 (r = −0.5742 and r = −0.2582, respectively), the correlation could be described as strong only in 2017. The amount of variability measured by the coefficient of determination (r^2^) was higher for the average air temperatures and average air humidity in June 2018, with r^2^ = 0.557 for the average air temperature and r^2^ = 0.6027 for the average air humidity) than for June 2017 (r^2^ = 0.3563 for the average air temperature and r^2^ = 0.3392 for the average air humidity) confirming that the weather conditions in 2018 were more favorable for ECB development than in 2017.

**Figure 1:**
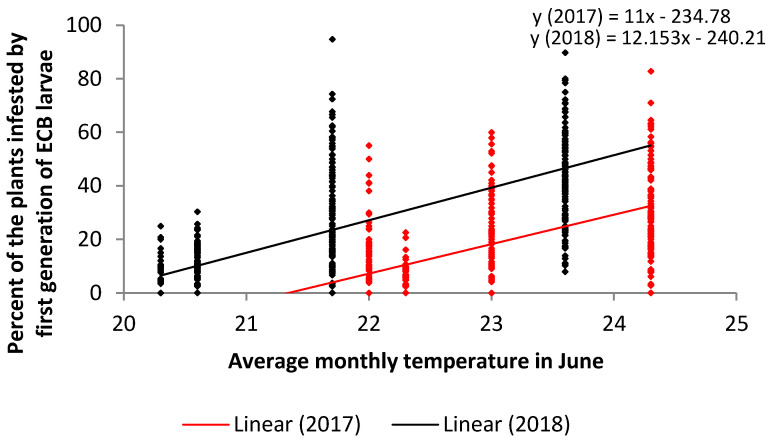
Regression analysis of the average monthly temperature in June (x) versus the percent of infestation with first generation of ECB larvae (y) in two years (2017—red and 2018—black).

The regression analysis performed for average monthly temperature in June (Figure 1) show that there is a linear growth in the percent of plants infested by the first generation of ECB larvae with the increase of average air temperatures in June from 20 °C to 24.5 °C.

**Figure 2:**
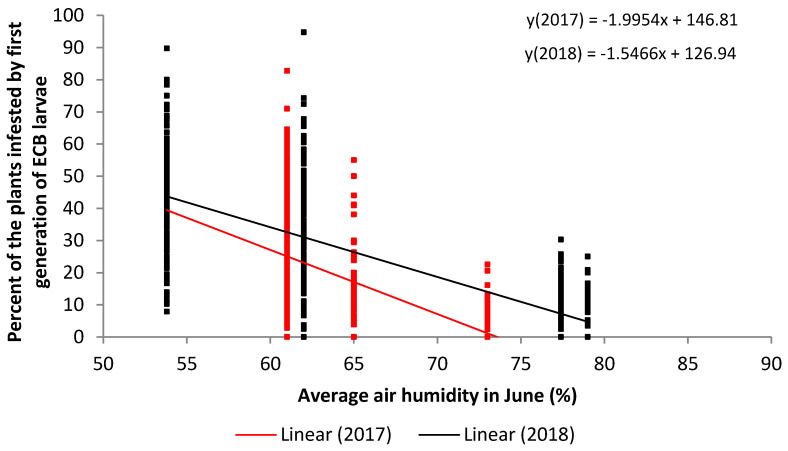
Regression analysis of the average air humidity in June (x) versus the percent of infestation with first generation of ECB larvae (y) in two years (2017—red and 2018—black).

The regression analysis performed for average air humidity in June (Figure 2) shows that there is a linear decrease in the percent of plants infested by the first generation of ECB larvae along with the increase of average air humidity in June from 50% to 80% relative air humidity.

## 3. Discussion

In the literature review, opposing data on the tolerance of different FAO maturity groups to ECB can be found. The tolerance is correlated with the agronomic and morphological traits of different FAO maturity groups rather than with any mechanism of the tolerance [30]. For example, Patch [35] reported that the height of maize or a factor, such as maturity correlated with height, was the main factor in the selection of maize by the ECB moths for oviposition. Maize hybrids planted earlier and the hybrids with extensive vegetative growth were attractive to moths to lay eggs, and therefore those hybrids suffered higher infestation from first generation ECB [25,32]. Recent investigations conducted by Leppik and Frérot [36] reported on maize odorscapes under field conditions that may improve host plant detection in ECB moths during oviposition.

To the best of our knowledge, this is the most comprehensive investigation of the Croatian market maize hybrids and the possible difference in their tolerance to ECB infestation. The insect pest resistance of hybrids on market is typically not declared. Thus, the research of tolerance to certain pests is a target of the research as is the case with Western corn rootworm (*Diabrotica virgifera virgifera* LeConte) not only in Croatia [37], but also in other neighboring countries [38] and at the general scale [39].

The results with Croatian hybrids and their tolerance to ECB reported by Ivezić and Raspudić [15], Lemić et al. [25] and Augustinović et al. [33] were based either on smaller number of hybrids or on the few locations included in the investigation. The lack of correlation between the FAO maturity group and the percent of infestation obtained in our study was in line with the data reported by Augustinović et al. [33], who reported significant differences in the intensity of the damaging effects on different locations and no significant differences concerning various hybrids. Similar results were obtained in the study with commercial maize hybrids in Poland [40], as well as with sweet corn [41] where it was shown that the percentage damage of the ECB larvae was different in each of the sampling plots and variety. In addition, the ECB larval damages were different for each of the sweet corn varieties, proving that the damage could not be correlated with hybrid, proving that locality and year had a major impact on the ECB attack.

The differences in the percent of attacked plants among hybrids have been established in the trials carried out at Šašinovec in 2017 and in Vrana in 2018 (Table 4 and Table 5). Based on the obtained results we cannot confirm that the strong and robust stem hybrids belonging to the later maturity group are more tolerant and do not suffer significant yield loss, in spite of the significant damage as reported by Lemić et al. [25] and Raspudić et al. [32]. In our study, we did not investigate the second generation attack and the yield loss; therefore, we cannot conclude on the tolerance to the yield loss.

Weather conditions were listed by many authors as the most significant factor influencing the appearance and intensity of ECB attacks [17,20,22,23,25]. Eclosion of the moths in Croatian conditions [14] is expected in May and egg laying occurs in May and in June, while egg hatching and larval development is expected in June. Therefore, we assumed that the weather conditions in May and June would be the critical for the first generation attack. Comparing the two years in which our investigation was performed, we observed that a significant difference was established between the years in the average air temperature in May and June.

The higher temperature was recorded in 2018 compared to 2017. Contrary to that, the average air temperature in June was higher in 2017 compared to 2018 (Table 1). Generally, in 2018, a higher total amount of rainfall was recorded compared to 2017; however, the difference between the years was not significant (Table 1). Among the locations, significant differences were established in the average air temperature in June and in the total monthly amount of rainfall in June (Table 2). The observed differences in weather conditions allow us to make conclusions regarding their impact on ECB attacks.

Many authors agree that weather conditions greatly influence ECB populations [42,43,44]. Rosca and Rada [22] reported on the positive impact of moderate air temperature and high air humidity on egg hatching and larval development. Barbulescou et al. [23] reported on the negative impact of high temperatures and drought resulting in high ECB larval mortality. The amount of precipitation in May and June and average air humidity in May and June were higher in 2018, comparing to 2017. Even though the differences were not significant, we can conclude that year 2018 was more favorable for ECB development than year 2017. This is confirmed by our results (Table 3).

The percent of plants infested on a single FAO hybrid at particular location (Table 4 and Table 5) ranged from 1.54% to 34.47% in 2017 and from 1.35% to 47.14% in 2018, respectively. The difference in the percent of plant infestation of all investigated maturity groups was established among locations in both years of investigation proving that weather conditions have major influence on the intensity of attack. Maize hybrids planted on locations in the mid part of Croatia, Šašinovec, and Gola, where the temperatures were lower and the amounts of rainfall were on average (but higher comparing to Vrana), recorded lower damages compared to Vrana, measuring higher temperatures in June in both years.

Intensive vegetative growth is a biological characteristic that attracts the first generation of ECB to intensifying their egg laying. However, the weather conditions are a crucial factor influencing the moth activity in June, as well as the egg laying, and egg hatching. Our results confirmed that the first generation attack of ECB was correlated with the weather conditions in June while the weather conditions in May were of less importance (Table 6). This is likely due to the fact that in Croatian conditions, oviposition and egg hatching took place in June [45].

According to many authors [42,43,44], oviposition and larval survival were reduced in years in which the temperatures or precipitations were below the average during the oviposition period. When the temperatures and precipitation were normal or above the average during oviposition, more ECB eggs were laid and the larval survival was higher. This was partially confirmed by our results as we established a strong to very strong correlation between average monthly temperature in June and the percent of attack intensity.

The regression line was linear and positive, which indicates that the percent of infestation increased with the increase of the average monthly temperature in June from 20 °C and 24.5 °C (Figure 1). Contrary to the statements that normal and increased precipitations in the oviposition period have a positive impact on larva development, our results did not prove a consistent impact of the total amount of rainfall in May and June on an increase or decrease of the percent of ECB attack intensity. Our results confirmed a negative impact of the increase of average air humidity (which is indirectly influenced by the amount of rainfall) in June on the percent of attacks in both years of investigation.

The regression line was negative and showed that the air humidity over 75% could be critical for larval development (Figure 2). Data presented by Showers et al. [46] implicate moisture (including inundation) and evaporation as especially potent factors in the suppression of the first and second instars of first generation ECB. However, it is difficult to compare our results because their observations were done under much higher temperatures (between 25 °C and 31 °C), and the moisture was expressed as moisture loss.

For egg laying and egg hatching, a warm and medium dry June is favorable. A high population level of the first generation, as we observed at Vrana and Tovarnik, may lead to a high level of second ECB generation as was reported by Lemić et al. [25], which ultimately caused yield reduction. In our investigation, we did not evaluate the attack of the second generation and did not compare the yield among hybrids. In the future, it would be interesting to evaluate the second generation as this can significantly increase the yield loss. However, establishing the yield loss and comparison among hybrids would be possible only between the same hybrids (untreated and treated with the complete protection against ECB).

## 4. Materials and Methods

### 4.1. Experimental Fields and Trial Design

Research was conducted in 2017 and 2018 at four locations in different climatic regions of Croatia: Šašinovečki Lug (45°51′00″ N, 16°10′01″ E; Central Croatia), Gola (46°1′44″ N, 16°33′13″ E; North-West Croatia), Tovarnik (45°13′28″ N, 19°21′38″ E; East Croatia), and Vrana (43°56′45″ N, 15°26′53″ E; Adriatic coast). Depending on the location, from 11 April until 5 May in 2017 and from 15 April until 6 May in 2018, in each of the four locations, 28 maize hybrids of Croatian breeding companies and four international hybrids belonging to four FAO maturity groups (300, 400, 500, and 600) were sown by row-column design in four replications. Every FAO maturity group was represented by eight commercially available hybrids (Table 7). In each group, one international hybrid and seven nationally developed and widely sown hybrids have been included. The hybrids were planted in four replication on 10 m^2^ plots (four 3.57 m long rows at row distance 0.7 m) with appropriate plant density.

### 4.2. Meteorological Data

Data collection on the average daily air temperature, daily amount of rainfall, and relative air humidity was done by setting up an automatic weather station (Davis 6250EU, Davis Instruments, Hayward, CA, USA) in the period between the first of May and the 30th of June in both years next to the maize fields at each location (Šašinovečki Lug, Gola, Tovarnik and Vrana).

### 4.3. Trial Assessments

The intensity of the first ECB generation attack was recorded between 28 June and 17 July 2017, as well as between 19 June and 11 July 2018. Within a plot all plants in the two inner rows of every replication, (i.e., 35–40 plants per replication or 160 plants per hybrid) were inspected on each location. The number of inspected and the number of damaged plants were recorded., only the number of Distinctive leaf holes and shot holes on stalks were identified as damage on the plants. The severity of symptoms was not recorded. The percent of attacked plants was calculated as a ratio of the number of attacked and the number of inspected plants.

### 4.4. Data Analysis

All data on the percent of infestation were compared between the FAO maturity groups, regions, and years by ANOVA by statistical software ARM 9^®^ [47], and the mean separation was estimated using Tukey’s honestly significant difference (HSD) test.

When required to correct skewness, the data were transformed using the *arc.syn x* or *√x + 5* transformation. Statistical software ARM 9^®^ [47] was used to calculate the correlation coefficients and to conduct regression analyses between the mean monthly air temperature, the total monthly amount of rainfall, and the average monthly air humidity as independent variables, and the percent of infestation as a dependent variable. The Pearson’s correlation coefficients were established, regression lines were described, and the coefficient of determination was calculated.

## 5. Conclusions

While intensive vegetative growth (often associated with hybrids belonging to later maturity groups) may attract ECB moths to lay eggs, and influence the attack of the first generation of ECB, the first generation attack was found to be primarily influenced by the weather conditions during the period of egg laying and hatching as well as during larval development. The first generation attack was positively correlated with the average air temperature in June within the range of 20 to 24.5 °C and was negatively correlated with the relative air humidity within the range of 50% to 80%. Our results provide a better understanding of the different factors influencing ECB damage. The obtained results could be useful for prediction of the damage from the first generation of ECB under the weather conditions similar to those observed in this research.

## Figures and Tables

**Table 1:** Comparison of the weather conditions (the average monthly temperature in °C, total amount of rainfall in mm, and average air humidity in %) in May and June between 2017 and 2018 and the result of statistical analysis.

Weather Indicator	Average Value ± SD for Year ^1^		HSD_*p* = 0.05_
	2017	2018	
Average monthly temperature in May (°C)	17.78 ± 0.83 b ^2^	19.45 ± 0.83 a	1.5
Average monthly temperature in June (°C)	22.90 ± 1.02 a	21.55 ± 1.49 b	0.752
Total monthly amount of rainfall in May (mm)	58.48 ± 12.95	103.60 ± 40.21	ns ^3^
Total monthly amount of rainfall in June (mm)	45.45 ± 24.99	118.20 ± 52.16	ns
Average air humidity in May (%)	68.00 ± 4.08	71.93 ± 5.91	ns
Average air humidity in June (%)	65.00 ± 5.66	70.23 ± 11.53	ns

^1^ Means and SD values are shown in original data units. ^2^ Means followed by the same letter in the same row are not significantly different (*p* = 0.05; Tukey’s honestly significant difference (HSD) test). ^3^ ns—not significant at *p* = 0.05.

**Table 2:** Comparison among the locations in weather conditions (the average monthly temperature in °C, total amount of rainfall in mm, and average air humidity in %) in May and June and the result of statistical analysis.

Weather Indicator	Average Value ± SD for Location ^1^				HSD_*p* = 0.05_
	Šašinovec	Tovarnik	Gola	Vrana	
Average monthly temperature in May (°C)	18.10 ± 0.71	19.10 ± 1.98	17.95 ± 1.48	19.30 ± 0.57	ns ^2^
Average monthly temperature in June (°C)	21.45 ± 1.20 b ^3^	22.35 ± 0.92 ab	21.15 ± 1.20 b	23.95 ± 0.49 a	1.613
Total monthly amount of rainfall in May (mm)	98.60 ± 80.75	80.15 ± 30.05	65.30 ± 10.89	80.10 ± 27.72	ns
Total monthly amount of rainfall in June (mm)	108.25 ± 69.65	99.65 ± 91.57	83.90 ± 16.83	35.50 ± 27.72	ns
Average air humidity in May (%)	75.00 ± 2.83	65.75 ± 3.18	71.35 ± 4.74	67.75 ± 6.72	ns
Average air humidity in June (%)	75.20 ± 3.11	65.85 ± 6.86	72.00 ± 9.90	57.40 ± 5.09	ns

^1^ Means and SD values are shown in original data units. ^2^ ns—not significant at *p* = 0.05. ^3^ Means followed by the same letter in the same row are not significantly different (*p* = 0.05; Tukey’s honestly significant difference (HSD) test).

**Table 3:** The average percent of plants (±SD) infested by European corn borer (ECB) larvae established on corn hybrids belonging to different FAO maturity groups in 2017 and 2018 and the results of statistical analysis.

FAO Maturity Group	2017	2018
FAO 300	14.13 ± 14.28 b ^1^	20.57 ± 20.89 ab
FAO 400	15.57 ± 14.65 b	18.95 ± 18.03 b
FAO 500	18.46 ± 16.77 ab	23.54 ± 22.00 a
FAO 600	20.27 ± 20.31 a	23.71 ± 22.94 a
HSD*_p_*_ = 0.05_^2^	4.525	4.487

^1^ Means and SD values are shown in original data units. ^2^ Means followed by the same letter within a column are not significantly different (*p* = 0.05; Tukey’s honestly significant difference (HSD) test).

**Table 4:** The average percent of plants (±SD) infested by European Corn Borer larvae established at four different locations and in four different regions in Croatia in 2017 and the results of the statistical analysis.

Locality	FAO Maturity Group				HSD*_p_*_ = 0.05_^3^
	300 ^1^	400 ^1^	500 ^1^	600 ^1^	
Šašinovec	1.54 ± 0.89 d ^2^ B ^3^	1.6 ± 0.97 d AB	3.76 ± 1.00 b A	1.99 ± 1.08 c AB	2.21
Gola	6.29 ± 1.73 c	8.14 ± 1.74 c	7.93 ± 1.52 b	9.87 ± 1.99 b	ns ^4^
Tovarnik	15.83 ± 1.55 b	17.43 ± 1.74 b	23.50 ± 1.47 a	23.58 ± 1.79 a	ns
Vrana	24.61 ± 1.50 a	27.02 ± 1.16 a	30.52 ± 1.71 a	34.47 ± 1.80 a	ns
HSD*_p_*_ = 0.05_^2^	3.44	3.69	4.54	4.99	

^1^ Data were transformed by using *arc.syn x* transformation. Means and SD values are reported in transformed data units and are not de-transformed. ^2^ Means followed by the same small letter within the columns are not significantly different (*p* = 0.05; Tukey’s honestly significant difference (HSD) test); small letters refer to differences among locations. ^3^ Means followed by the same capital letter within the rows are not significantly different (*p* = 0.05; Tukey’s honestly significant difference (HSD) test); capital letters refer to differences among hybrids. ^4^ Not significant.

**Table 5:** The average percent of plants (±SD) infested by European corn borer larvae established at four different locations and in four different regions in Croatia in 2018 and the results of the statistical analysis.

Locality	FAO Group				HSD*_p_*_ = 0.05_^3^
	300 ^1^	400 ^1^	500 ^1^	600 ^1^	
Šašinovec	6.33 ± 1.17 c	8.56 ± 1.04 c	8.39 ± 0.95 c	8.78 ± 1.15 c	ns ^4^
Gola	1.35 ± 1.00 d	1.68 ± 1.09 d	2.21 ± 1.09 d	1.36 ± 1.20 d	ns
Tovarnik	27.03 ± 1.95 b	25.03 ± 1.69 b	31.21 ± 1.84 b	30.22 ± 1.66 b	ns
Vrana	39.97 ± 1.29 a AB	33.97 ± 1.27 a B	45.59 ± 1.22 a A	47.14 ± 1.43 a A	9.85
HSD*_p_*_ = 0.05_^2^	3.36	3.05	3.50	3.26	

^1^ Data were transformed by using *arc.syn x* transformation. Means and SD values are reported in transformed data units and are not de-transformed. ^2^ Means followed by the same small letter within the columns are not significantly different (*p* = 0.05; Tukey’s honestly significant difference (HSD) test): small letters refer to differences among locations. ^3^ Means followed by the same capital letter within the rows are not significantly different (*p* = 0.05; Tukey’s honestly significant difference (HSD) test); capital letters refer to differences among hybrids. ^4^ Not significant.

**Table 6:** The correlation coefficients and coefficients of determination for ECB infestation expressed as a % of the attack of first generation as a dependent variable on different weather conditions (the mean air temperature, total amount of rainfall, and average air humidity) as independent variables in two years of investigation.

Independent Variable	Month	Year	*n*	Correlation Coefficient r	Coefficient of Determination r^2^	*p*	Type of Correlation
Mean air temperature	May	2017	512	0.48	0.2375	0.0001	medium
	May	2018	512	0.57	0.3274	0.0001	strong
	June	2017	512	0.59	0.3563	0.0001	strong
	June	2018	512	0.74	0.5570	0.0001	strong
Total amount of rainfall	May	2017	512	0.23	0.0574	0.0001	very weak
	May	2018	512	0.03	0.0014	0.0405	not existing
	June	2017	512	−0.57	0.3298	0.0001	strong
	June	2018	512	−0.25	0.0667	0.0001	weak
Average air humidity	May	2017	512	−0.59	0.3567	0.0001	strong
	May	2018	512	0.14	0.0225	0.0007	very weak
	June	2017	512	−0.58	0.3392	0.0001	strong
	June	2018	512	−0.77	0.6027	0.0001	very strong

**Table 7:** List of the maize hybrids involved in the investigation.

FAO 300		FAO 400		FAO 500		FAO 600	
Hybrid	Company	Hybrid	Company	Hybrid	Company	Hybrid	Company
**Bc 344**	Bc ^1^	Os 444	Os	Os 552	Os	Bc 682	Bc
**Bc 323**	Bc	Bc 406	Bc	Bc 525	Bc	Bc 616	Bc
**Bc 306**	Bc	Bc 424	Bc	Bc 575	Bc	Bc 626	Bc
**TRIO**	Bc	Bc 482	Bc	Klipan	Bc	Riđan	Bc
**P9903**	DuPont ^2^	DKC 4608	DeKalb ^4^	DKC 5830	DeKalb	P1535	DuPont
**Os 378**	Os ^3^	Kulak	Os	Velimir	Os	Rudolf 60	Os
**Os 398**	Os	Tomasov	Os	Os 5922	Os	Os 6217	Os
**Os 3617**	Os	Drava 404	Os	Os 515	Os	Os 635	Os

^1^ Bc—Bc Institute Rugvica, Dugoselska 7, Croatia. ^2^ DuPont—Corteva Agroscience, Chestnut Run Plaza 735, Wilmington, DE, 19805-0735, USA. ^3^ Os Agricultural Institute Osijek, Južno predgrađe 17, Osijek, Croatia. ^4^ DeKalb—DeKalb Genetics Corporation, 3100 Sycamore Rd, DeKalb, IL 60,115 United States.
